# Left Ventricular Motion Analysis Framework for the MATRIX-VT Study

**DOI:** 10.1007/s11548-025-03510-1

**Published:** 2025-09-05

**Authors:** Christian Janorschke, Sorin S. Popescu, Jonas Osburg, Xinyu Lu, Jingyang Xie, Engin Yaman, Christoph Marquetand, Oliver Blanck, Hannes Alessandrini, Achim Schweikard, Roland R. Tilz

**Affiliations:** 1 Institute of Robotics and Cognitive Systems, University of Lübeck, Ratzeburger Allee 160, Lübeck, 23652 Germany; 2 Department of Rhythmology, University Heart Center Lübeck, University Hospital Schleswig-Holstein, Ratzeburger Allee 160, Lübeck, 23652 Germany; 3 German Center for Cardiovascular Research (DZHK), Partner Site Lübeck, Lübeck, Germany; 4 Medical Clinic 2, University Heart Center Lübek, University Hospital Schleswig-Holstein, Lübeck, Germany; 5 Department of Radiation Oncology, University Medical Center Schleswig-Holstein, Kiel, Germany

**Keywords:** Left ventricular motion, 3D echocardiography, Ultrasound and computed tomography fusion, Stereotactic arrhythmia radioablation

## Abstract

****Purpose**:**

Ultrasound (US) is commonly used to assess left ventricular motion for examination of heart function. In stereotactic arrhythmia radioablation (STAR) therapy, managing cardiorespiratory motion during radiation delivery requires representation of motion information in computed tomography (CT) coordinates. Similar to conventional US-guided navigation during surgical procedures, 3D US can provide real-time motion data of the radiation target that could be transferred to CT coordinates and then be accounted for by the radiation system. A motion analysis framework is presented that covers all necessary components to capture and analyse US motion data and transfer it to CT coordinates.

****Methods**:**

Utilizing a robotic test set-up with a human phantom, a baseline and ground truth dataset is recorded for the development and implementation of the motion analysis framework. An optical tracking system and an additional spatial calibration phantom are used to determine necessary transformations. Methods for frame matching, calibration, registration and evaluation are implemented.

****Results**:**

The hardware set-up meets all requirements, including a frame rate exceeding 20 Hz and acceptable image quality, while involving only a few components that can easily be mounted and dismantled in a clinical context.The recorded phantom dataset meets all hardware-specific requirements including a frame rate exceeding 20 Hz, an offset between CT trigger time and the closest US recording of 2–20 ms as well as acceptable US image quality. The static phantom allows for quantitative evaluation by matching structures from different US frames in CT coordinates. While each individual step of the US and CT fusion process achieves the target accuracy of less than 5 mm error, the cumulative error over all transformations exceeded this limit for extreme probe positions.

****Conclusion**:**

The framework is developed and tested for the MATRIX-VT study and can be utilized for patient data evaluation as well as for transferring information such as positional data of moving anatomical structures between US and CTpredictive motion management in STAR therapy. Its modular design allows for the incorporation of advanced calibration and registration methods to address probe positioning limitations, thereby enhancing overall system performance for future applications.

## Introduction

Left ventricular (LV) motion is usually analysed for heart function assessment and detection of abnormalities [1, 2]. Recent research has focused on automatic myocardium detection and segmentation to calculate metrics like ejection fraction and strain rate [3, 4]. These approaches could also be considered for patient-specific motion management for stereotactic arrythmia radioablation (STAR) therapy [5]. This therapy treats ventricular tachycardia by delivering radiation dosages to a target volume in the myocardium. The target is defined similar to regular ablation and then transferred to computed tomography (CT) for radiation planning. Considering the amount of critical structures in close proximity (valves, oesophagus, lung ...) and the combined motion of both respiration and cardiac pumping, research of motion management strategies aims to decrease the risk of long-term complications either by lowering the necessary dosage due to increased precision or by protecting critical structures. Presently, strategies like breath hold, gating and abdominal compression are deployed to deal with respiration, but the development of combined cardiorespiratory motion management strategies is ongoing and recommended by current research [6–8]. Due to many advantages of 3D echocardiography, like being real-time capable, low-cost and radiation free, we propose an ultrasound (US)-based strategy. Theoretically, this enables live motion management: Similar to conventional US-guided treatments, motion from the probe and object-tracking in US data can be combined to capture cardiac and respiratory movement. Afterwards, the information can be transferred to CT coordinates with an underlying registration and then be accounted for by the radiation system. To create a database for the development of such a system, the MATRIX-VT study (New 3D-echocardiography-based Motion integration and mAnagement for subsTrate characteRisation and stereotactic arrhythmia radIoablation tX in Ventricular Tachycardia) was designed in 2023. Its primary objective is the development of software capable of capturing LV motion in 3D US and mapping it to CT coordinates, thereby demonstrating the feasibility of US-based motion management for applications such as STAR therapy. Therefore, patients with ventricular arrhythmia undergoing catheter ablation procedures with pre-procedural cardiac contrast CT are included. A test dataset is recorded beforehand using a human phantom. Uncontrollable motion by both patient and physician are eliminated with use of robotic US, thereby creating a ground truth dataset without displacement and deformation that can be used for numeric evaluation. This paper describes the development and evaluation of a framework to collect and transfer motion data in US and transfer it to CT coordinates based on a robotic test set-up for the MATRIX-VT study. We limited the software to state-of-the-art algorithms that can later be refined or exchanged if needed.

## Methods

**Fig. 1:**
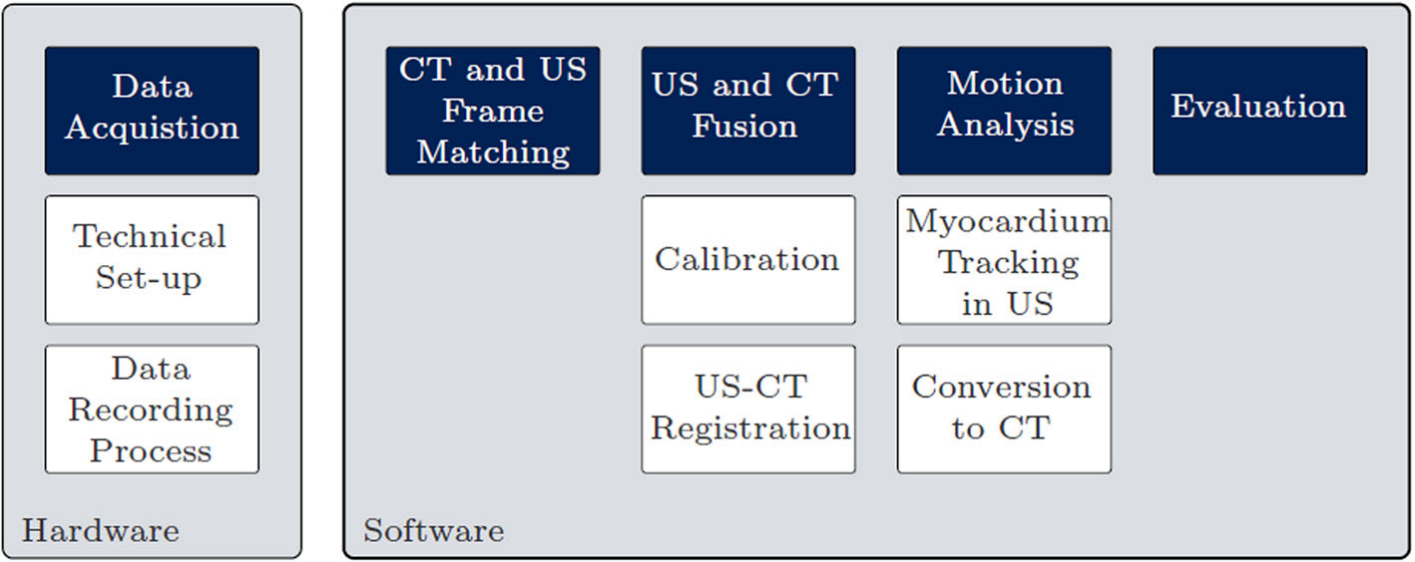
Components of the motion analysis framework

The objective of this work is the design of a modular framework for LV motion analysis. Hence, the hardware, process of data acquisition and evaluation methods have to be defined. Figure 1 gives an overview of all components of the motion analysis framework.

On the hardware side, there is the technical set-up and the process of data recording. On the software side, six sub-steps are labelled: CT and US frame matching, calibration, US-CT registration, myocardium tracking in US, conversion to CT and evaluation. After acquiring and preprocessing the data, it will be analysed following these six steps. Single functions can easily be exchanged for adjusted or improved versions.

### Robotic test set-up

**Fig. 2:**
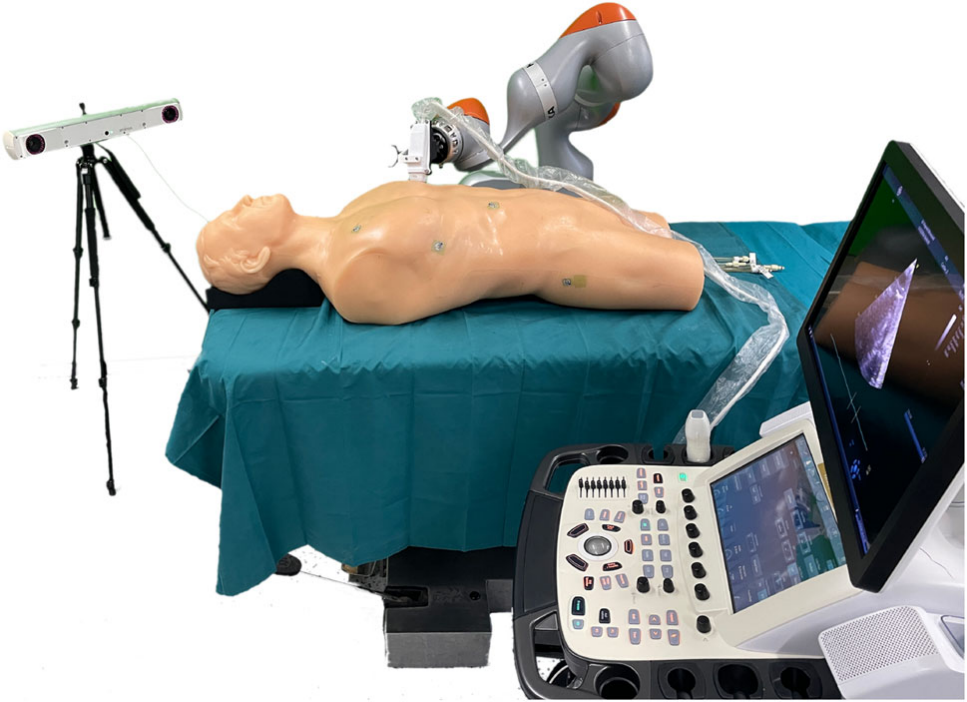
The robotic set-up for capturing optical tracked 3D ultrasound data of a human phantom

To prove functionality and to create a baseline dataset, the set-up is tested with a human phantom. This dataset has no cardiorespiratory motion and can therefore function as the evaluation baseline. After transferring the myocardium segmentations from different probe positions to CT coordinates, all displacements can be interpreted as errors. Figure 2 shows the laboratory set-up.

The US examination is performed with the Blue Phantom FAST Full-Torso phantom in the supine position, equivalent to a CT scan. An apical probe position is chosen to capture the apex, mitral valve and aortic valve. The US probe is mounted on the end effector of a KUKA LBR iiwa 14 R820 robot using a 3D printed probe holder, specifically designed for the 4Vc-D probe of the US station Vivid E95 from GE HealthCare. Four attached optical markers are tracked by the Atracsys fusionTrack 500, positioned approximately 1.5 m away. During each recording, the tracking data, the robot positions, the ECG signal recorded by the US station and the US volumes are streamed via a local area network and stored with time stamps. The full US recording is then archived for backup. Receiving time stamps in ECG and tracking data allows for temporal synchronization. Additionally, the US data is synchronized with ECG data via station time stamps to avoid network delays from transmitting 3D US volumes. This way, the transmission of 3D US data does not affect the temporal error, because the volumes are synchronized to the one-dimensional ECG samples with a high sampling rate (600 Hz). The optical tracking is transmitted via the plus toolkit [9] in form of tracking matrices rather than a video stream to minimize latency. The initial probe position is aligned manually in compliance mode. The electrocardiogram (ECG) of the (healthy) operator defines the length of ten cardiac cycles. A manual US examination in compliance mode over ten cardiac cycles of the healthy operator is used to define a motion trajectory for the robot. Variance in the US data and probe positions is introduced by applying extreme translations and rotations to the probe, while ensuring that the LV remains visible on the display. By copying the trajectory and returning to the initial position at the end, continuous motion cycles are created. Positional information is saved in form of joint position matrices. Since one free breathing and one dataset under breath hold are planned per patient, two datasets of ten cardiac cycles each are recorded. In context of the human phantom, the second dataset represents a test of repeatability and is merely stored as backup.

The frame rate is kept at a minimum of 20 Hz. This should suffice, considering common heart rates of about 1–2 Hz leading to upwards of ten US volumes per heartbeat, while keeping acceptable image quality. Also, this limits the maximum error in time between the diastolic US frame and the CT trigger to 25 ms (half of 50 ms interval). This error results from both frame rate limitations of the US station and heart rate fluctuation [10]. The remaining station settings are optimized for image quality and kept constant for both recordings. For spatial calibration based on the work of von Haxthausen et al. [11], a third dataset is recorded with identical US station settings utilizing a spatial calibration phantom with optical markers by manually aligning the probe and translating and rotating it in compliance mode of the robot. For the clinical setting, a short manual US scan of the calibration phantom is sufficient, provided that the inner structures and the optical markers of both probe and phantom are visible in US and the tracking data respectively.

Recording a calibration dataset after each patient examination with identical settings allows for patient-specific US settings and thus enhanced image quality and acceptance from the conducting physician, since the settings can be optimized by the physician before the recording. Resulting changes in spacing, size and orientation of the US volumes are afterwards handled by the calibration process that is based on the known geometry of the calibration phantom.

### CT and US frame matching

The CT protocol of the MATRIX-VT study defines the CT trigger percentage as 78% R-R interval. For the correct US to CT transfer, a best matching US volume has to be determined. Therefore, the R-peaks are extracted from the ECG data by a local maximum search to determine the closest recorded time to the CT trigger per interval. The best fit of ten recorded cycles is determined for each dataset to avoid the maximum possible error of 25 ms caused by the US framerate. The resulting US frame and the CT are segmented using the software 3D Slicer [12]. Additionally, the apex and the centres of the mitral and aortic valves are annotated. These landmarks are later used for pre-alignment between US and CT.

### US and CT fusion

**Fig. 3:**
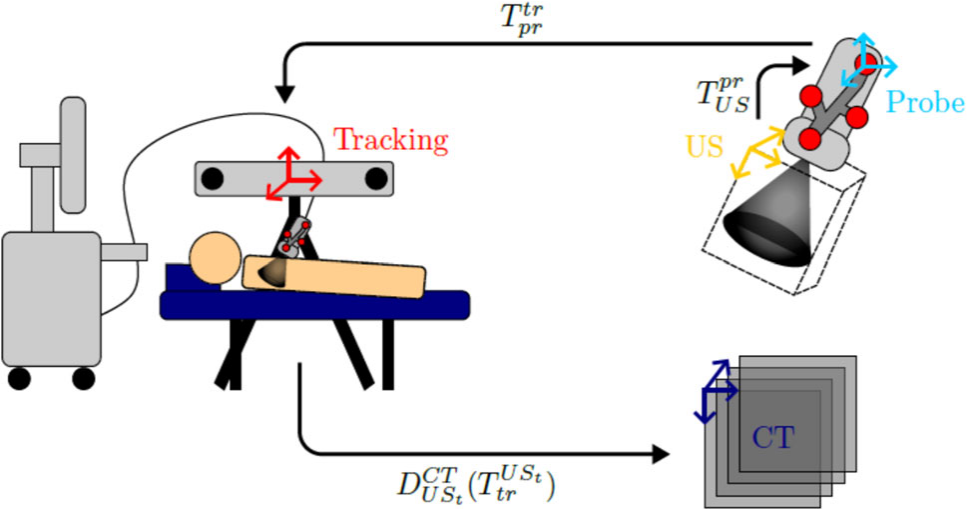
Forward calculation from ultrasound to computed tomography coordinates

For the fusion of US and CT, an equation is needed to transfer any point in US to CT space while being handed through the tracking space to deal with movement of the probe or patient. Figure 3 visualizes this process.

A voxel first has to be mapped to the US spacing ($A^{US}_{UsVox}$), then transformed to the probe coordinate system ($T^{pr}_{US}$), then transformed to the tracking system ($T^{tr}_{pr}$) and finally moved to CT based on a 3D/3D registration of the best match between US and CT ($D^{CT}_{US_t} ( T^{US_t}_{tr} )$). Any point can thus be expressed as:

1 $$\begin{aligned} p^{CT} = D^{CT}_{US_t} ( T^{US_t}_{tr} \cdot T^{tr}_{pr} \cdot T^{pr}_{US} \cdot A^{US}_{USVox} \cdot p^{UsVox}) \end{aligned}$$

This equation has to be established for every dataset. While the spacing and tracking matrices are given, the transformations $T^{pr}_{US}$ and $D^{CT}_{US_t} ( T^{US_t}_{tr} )$ have to be determined.

#### Calibration matrix

$T^{pr}_{US}$ is the transformation from US space to the coordinate system defined by the optical markers. It is the result of the calibration process following the approach of von Haxthausen et al. [11]. The calibration was further refined and automated for this work. It relies on the inner structures of the spatial calibration phantom that are matched to its CT scan, so that the missing transformation can be expressed as:

2 $$\begin{aligned} T^{pr}_{US} = T^{pr}_{tr} \cdot T^{tr}_{CT} \cdot T^{CT}_{US} \end{aligned}$$

**Fig. 4:**
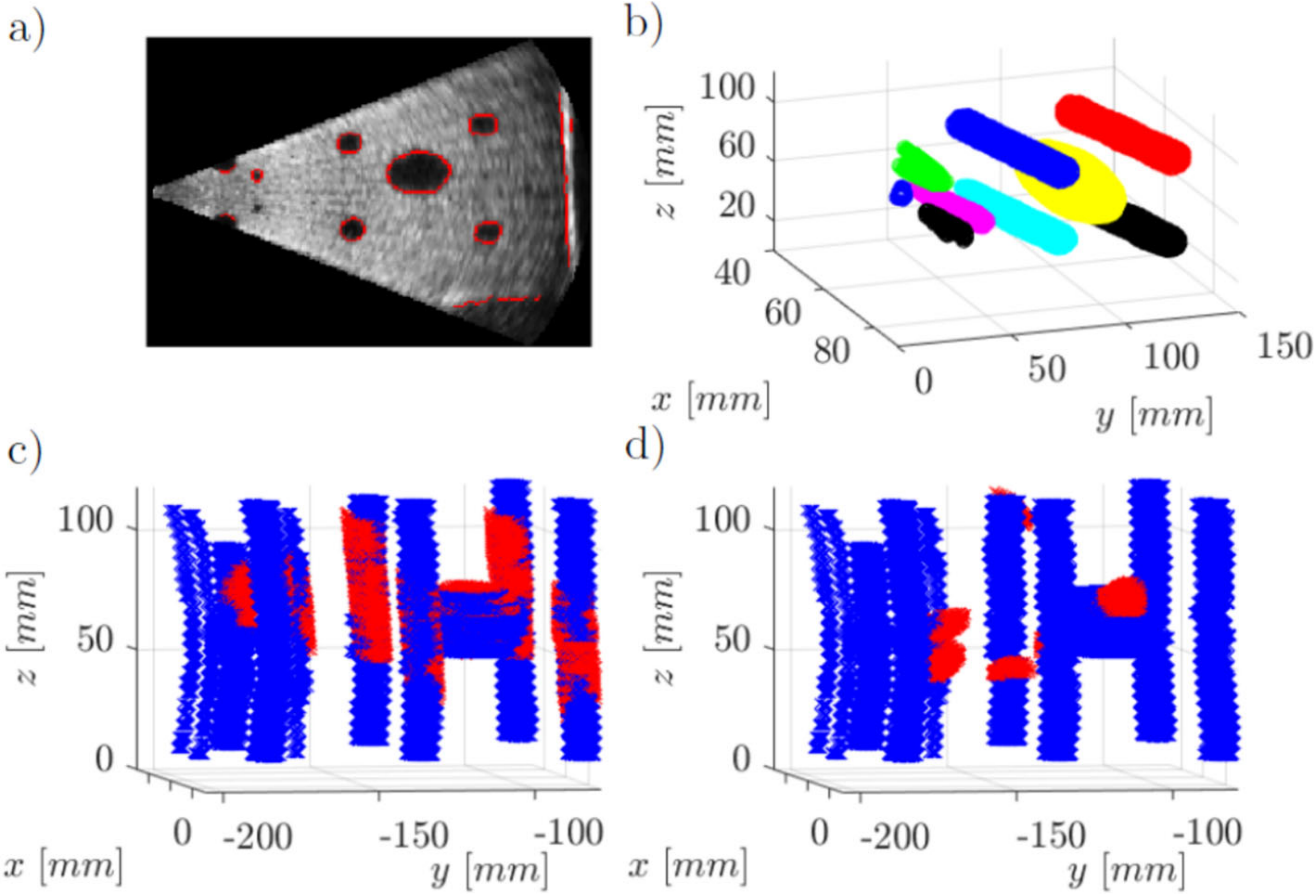
Process of spatial phantom calibration: a) Segmentation b) clustering (represented by colors) c) successful registration d) failed registration (Blue: computed tomography segmentation, red: registered ultrasound segmentation)

The transformation $T^{tr}_{CT}$ is determined using the optical markers in tracking and CT space. For CT and US matching, the inner structures have to be segmented, pre-aligned and registered to the manual segmentation of the CT scan. The automated process consists of: edge-based segmentation with the Canny algorithm (Fig. 4a), post-processing to adjust for the US cone, clustering using the dbscan algorithm (Fig. 4b), pre-alignment and finally registration using the iterative closest point algorithm (Fig. 4c and d).

#### Registration from US to CT

The second unknown variable for the US and CT fusion is the transformation from the matched US frame to the CT $D^{CT}_{US_t} ( T^{US_t}_{tr} )$. It is expressed as a point-wise deformation after a rigid pre-alignment to capture time related or positional changes from CT to US examination. The matrix is the result of a 3D/3D registration between two segmentations of the myocardium of the LV. Here, the annotations of the valves and apex are used for pre-alignment. The coherent point drift algorithm is used to determine the affine registration. Registration results improved when including normalization before registration. To further increase precision and to deal with delays in time stamps between tracking information and US data caused by differences in frame rates of the device, the tracking matrices are interpolated using spherical linear interpolation between quaternions.

### Motion analysis

With Eq. 1, any point in US coordinates can be transferred to CT coordinates. To prove feasibility of the motion analysis framework, the myocardium of different US volumes can be transferred to CT coordinates. Theoretically, these pointclouds match in the CT coordinate system due to the phantom’s static LV. The detection of the myocardium in the recorded US frames can be implemented in several ways, the simplest of which is manual segmentation in frames of interest, like frames corresponding to maximum robot movement. In later stages, algorithms for detection, registration or tracking in consecutive US frames can be implemented for detailed motion analysis. This part of the motion analysis framework and its optimization is most dependent on actual patient data. Hence, five frames are manually segmented and the resulting pointclouds are afterwards transferred to CT coordinates.

### Methods for evaluation

The motion analysis of phantom and patient data requires evaluation and visualization. The 17-segment model is chosen for a uniform visual representation [13]. It can visualize overall displacement or be split in either x, y and z or radial, longitudinal and rotational displacement diagrams. The tools and alignment of the model are based on Janorschke et al. [14]. Specific substructures from all US frames of a cardiac cycle can also be superimposed in CT. This is useful for instance in radiation planning, to avoid radiating specific structures or volumes. In this work, all LV pointclouds should match in CT coordinates. Hence, the calculation of metrics for pointcloud comparison like Hausdorff distance (HD) or DICE score can be used for assessment of the total registration error. Since US recordings might crop some part of the myocardium, unidirectional metrics from US to CT better represent registration quality.

## Results

To prove the functionality and feasibility of the developed motion analysis framework, each step is evaluated independently before assessing the overall resulting error.

### Calibration with spatial calibration phantom

For calibration consistency assessment, the process is performed 100 times. The unidirectional HD of the inner structures after alignment ranged from 3.8 to 29.6 mm. The calibration matrices of the 30 frames with lowest HD are chosen to automatically and objectively filter out calibration attempts in which probe positioning or contact force negatively affect the image quality. In these cases, the registration fails due to missing information of the inner structures. This effect can be seen in Fig. 4d. In clinical settings, inspection of the frames chosen for calibration prevents this kind of failure without requiring long calibration recordings and multiple calculations of registrations and pointcloud metrics.

**Fig. 5:**
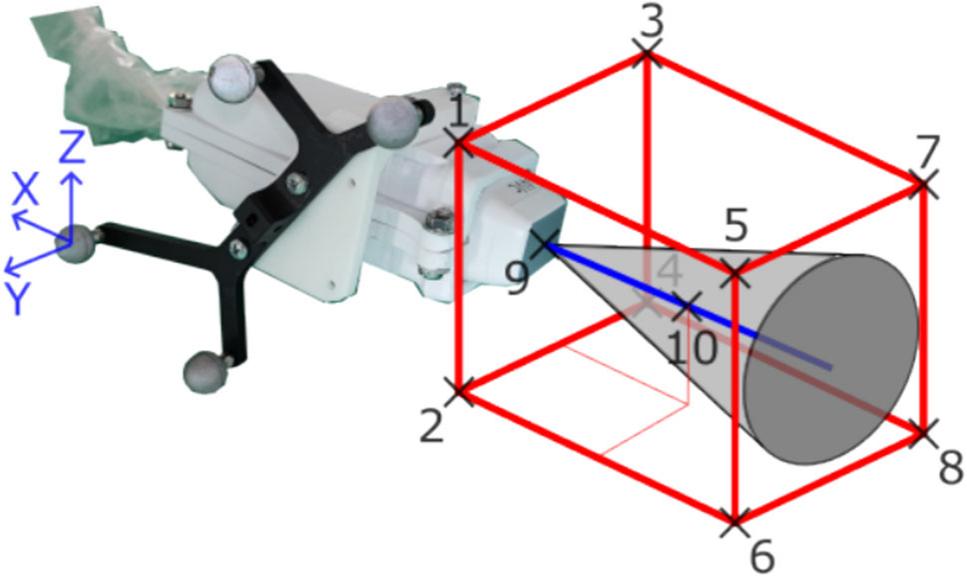
Positions and orientation of calibration consistency assessment

With these calibration matrices, ten points are transferred into the probe coordinate system: Fig. 5 visualizes the positions and orientation.

The maximum combined variance of 5.0 mm resulted from transformation of point 7, due the highest distance to the probe coordinate system origin. Point 9 and point 10 have combined variances of 3.3 mm and 3.4 mm respectively, showing less variation in the regions of interest. The standard deviations are listed in Table 1.

**Table 1:** Standard deviations for ten evaluated positions in mm (positions shown schematically in Fig. 5)

Point	1	2	3	4	5	6	7	8	9	10
$\sigma _x$	0.6	1.2	1.5	1.9	1.0	2.0	1.9	2.5	1.1	1.3
$\sigma _y$	1.5	1.7	2.0	1.6	1.8	1.5	2.6	2.1	1.2	1.4
$\sigma _z$	4.0	2.0	3.9	1.9	3.9	2.3	3.8	2.2	2.9	2.8

Besides the calibration consistency, calibration accuracy can be assessed by evaluating the transfer of landmarks into tracking space over multiple frames. Therefore, the centre of the spatial calibration phantom is chosen and manually annotated in 20 consecutive frames. The probe is simultaneously rotated by 2, 15 and 45 degrees around the x, y and z axes in the tracking system and translated by 7.1 mm over these frames, resulting in high image variance. A ground truth position in tracking space is determined by transferring the CT coordinate of the landmark into tracking space. This allows for simultaneous evaluation of the tracking system by repeating the CT coordinate transfer with the recorded tracking matrices. Table 2 lists the results of the landmark transfer.

**Table 2:** Landmarks in tracking space. *x*, *y* and *z* coordinates in mm for 20 transfers of computed tomography ground truth landmark and consecutive ultrasound annotations

Coordinate	$x^{CT}_{Tr}$	$x^{US}_{Tr}$	$y^{CT}_{Tr}$	$y^{US}_{Tr}$	$z^{CT}_{Tr}$	$z^{US}_{Tr}$
Mean	501.2	501.1	147.9	149.4	1462.5	1464.6
Median	501.2	502.0	147.9	149.6	1462.5	1463.7
Min	501.2	494.3	147.8	147.7	1462.5	1459.5
Max	501.5	506.6	148.0	151.1	1462.6	1470.7
Std.	0.08	3.72	0.04	1.08	0.03	3.85

### US and CT registration

The US and CT registration for calibration purposes is based on manual segmentations. While the CT segmentations can be assumed to be highly accurate, manual US segmentations come with higher inter- and intra-observer variability. Hence, five out of 179 recorded ultrasound volumes were segmented twice by the same operator for evaluation purposes. The US volumes correspond to highest probe position variability determined from the tracking trajectory to avoid strong similarities in the US data. The segmentations of corresponding frames have a median DICE similarity of 0.85, a median bidirectional HD of 7.6 mm and a respective 95 percentile (HD95) of 1.4 mm. After segmentation, the myocardium is registered and then compared to the CT segmentation. The results listed in Table 3 describe the quality of the affine registration. Minimum and maximum HD95 were 3.0 and 4.5 mm respectively with a median value of 3.7 mm.

**Table 3:** Results of the myocardium 3D/3D registration from ultrasound to computed tomography: Unidirectional Hausdorff distance and the 95 percentile in mm for two segmentations each

Frame	44/1	44/2	97/1	97/2	105/1	105/2	123/1	123/2	154/1	154/2
HD	10.7	10.8	12.2	12.2	10.2	10.8	13.0	12.1	9.4	10.2
HD95	4.5	3.7	3.1	3.0	3.6	3.1	3.4	3.8	4.4	4.1

Notation: The first segmentation of frame 44 is labelled 44/1

### Motion analysis in CT coordinates

The cumulative error starting from calibration and ending with the transfer of motion data from US to CT can be assessed using the same segmented volumes. This time, the segmentations are not directly registered onto the CT segmentation but instead follow the forward calculation given in Eq. 1. Each of the US segmentations is used as the corresponding US frame to the CT scan once. The remaining segmentations are then transferred to CT via the optical tracking data. Table 4 lists the results.

**Table 4:** Phantom myocardium transfer from different ultrasound (US) frames and probe positions to computed tomography (CT) coordinates

Frame	44/1	44/2	97/1	97/2	105/1	105/2	123/1	123/2	154/1	154/2
44/1	4.5	4.4	13.1	13.6	7.2	7.0	6.9	6.3	5.6	5.5
44/2	4.2	3.7	13.6	14.0	8.2	7.7	8.0	7.1	4.7	**4**.**6**
97/1	20.2	**21**.**9**	3.1	3.7	9.3	8.3	10.4	11.8	20.5	21.5
97/2	18.6	19.9	3.2	3.0	8.1	7.3	9.2	10.8	18.7	19.8
105/1	11.3	12.6	8.1	8.3	3.6	3.0	3.6	4.5	11.5	12.3
105/2	12.5	13.7	8.5	8.7	4.3	3.1	4.3	5.5	12.5	13.5
123/1	9.9	10.9	8.4	8.6	4.1	3.9	3.4	4.0	10.4	10.8
123/2	8.7	9.6	9.3	9.6	4.1	3.5	3.6	3.8	9.1	9.3
154/1	4.8	4.6	14.7	15.4	9.1	8.5	8.4	7.7	4.4	4.0
154/2	5.6	5.1	15.4	16.2	9.9	9.0	9.3	8.5	4.7	4.1

Unidirectional 95 percentile Hausdorff distance in mm from transferred US to CT ground truth segmentation. Columns: tracking frames that are transferred to CT, rows: registration baseline frame. Notation: The first segmentation of frame 44 is labelled 44/1

**Fig. 6:**
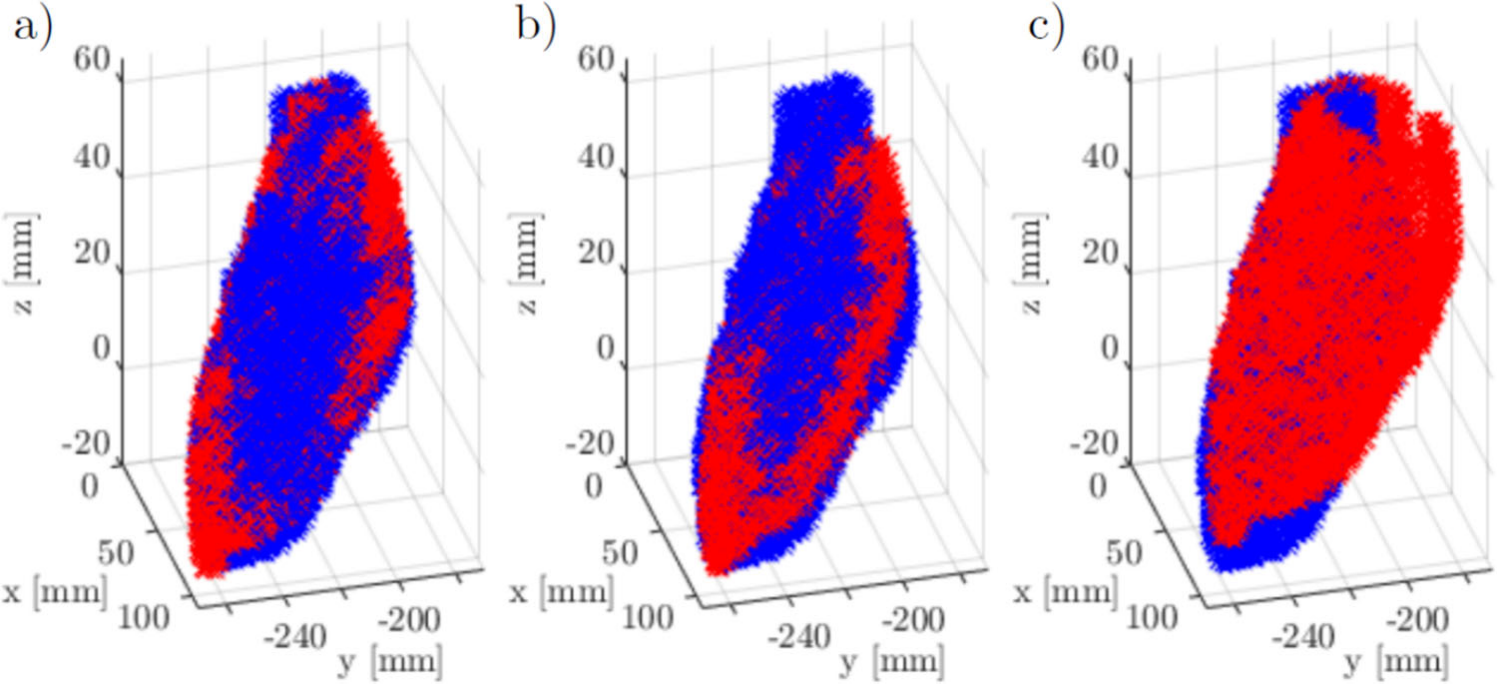
Overlay of computed tomography ground truth (blue) and transferred ultrasound myocardium segmentation (red), best and worst case for separate frames: a) direct registration frame 44 b) transferred segmentation of frame 154 based on frame 44 c) transferred segmentation of frame 44 based on frame 97

Figure 6 shows the best and the worst conversion in form of overlapping 3D volumes. The best and the worst case of separate frames are compared to the direct registration of frame 44. All three scenarios show a good overlap at the apex, but increasingly worse performance in the aortic regions. Manipulation of the perspective reveals the rotational errors around the *y* and *x* axis that result in the domination of red points in Fig. 6c. The numeric result for both cases is highlighted in Table 4.

**Fig. 7:**
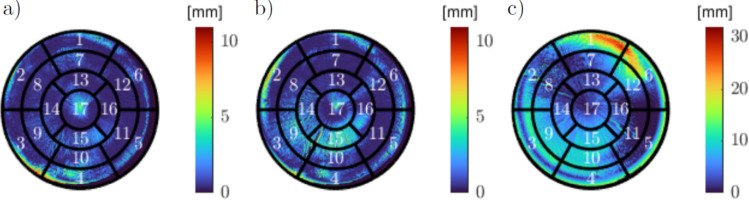
17-segment representation of displacement after myocardium transfer. Maximum value in radial direction: a) direct registration frame 44 b) transferred segmentation of frame 154 based on frame 44 c) transferred segmentation of frame 44 based on frame 97 (aortic valve is positioned in centre of segment 2)

Additionally, the 17-segment visualization of these cases is given in Fig. 7 by calculating the closest distance to a segmented voxel in CT for all transferred points. Afterwards, the maximum displacement of radial wall segments is determined to plot the displacement over the LV in the 17-segment bullseye view.

## Discussion

The developed motion analysis framework shows promising results: On the hardware side, US volumes can be streamed with more than 20 Hz frame rate and individual settings for optimized image quality.

The optical tracking set-up consists of the tracking camera and a tripod, and is therefore easy and fast to mount and dismantle. Inserting the US probe into the 3D printed probe holder is the only necessary preparation on the US side. A router is used for the local area network connection, allowing quick and simple set-up of all hardware components. The actual recording time was less than a minute for all datasets. The time required for explanations, patient preparation, optimization of station settings and probe positioning has yet to be determined for human patients. Nevertheless, the phantom test indicates feasibility of the proposed methods for passive motion management in STAR therapy, since data preprocessing and analysis can be performed after data acquisition, similar to the current work flow for CT segmentations in STAR therapy. However, a real-time capable motion management system in the future will require (semi-)automatic segmentation of the myocardium. Overall, theThe process of data recording is repeatable, fast and flexible regarding the positions of the tracking system, US station, and physician. Therefore, it will be repeated for the MATRIX-VT patients in the clinic. On the software side, the calibration and the CT registration process show accuracies in the 5 mm range when evaluated independently, while the overall combination of all methods performed worse for extreme probe positions. While this kind of probe motion is not expected to be caused by the patient’s respiration, calibration and registration should be refined with more advanced algorithms. Allowing scaling and shearing in the calibration process is likely to improve the results, due to the spatial resolution of US being influenced by contact force and surface angle. Similarly, a non-rigid registration between US and CT may be necessary address these effects and changed conditions between CT and US examination. Figure 7 a and b highlights the regions of highest displacement, particularly around the aorta and towards the left atrium. Since additional inter-observer variability in US segmentations further affects these results, the patient data should only be segmented by trained physicians and follow a uniform definition of the border between LV and aorta or atrium. Still, a HD95 of less than 5 mm was achieved in multiple cases and for all repeated segmentations, suggesting acceptable accuracy for minor probe motion in between frames. The actual motion analysis has been included in form of manual segmentation of the myocardium. Theoretically, the presented work could already be used to aid radiation planning by visualization of structures during systole in the diastolic planning CT. With the implemented methods, segmented systolic structures can be transferred to CT taking differences in probe position into account. To provide the actual displacement of the segmentation, a frame-by-frame approach for the tracking of the myocardium will be added to the framework. Current research on motion management for STAR therapy highlights various approaches. The most common methods include gating, breath hold and abdominal compression. Recently, magnetic resonance imaging and US guidance techniques have also been explored [7, 8, 15]. Additionally, Perrin et al. [16] proved the feasibility of tracked US image acquisition during cardiac radioablation. Tracking accuracies of different motion management strategies have not been systematically evaluated to date. Consequently, assessments involving a moved probe on a static phantom or cross-evaluations with actual patient data could provide valuable insights.

All in all, the robotic set-up is a successful test for the MATRIX-VT study which is conducted with the same hardware and can be evaluated using the developed motion analysis framework. Due to its modular approach, further improvements and functionalities can easily be integrated. Some are already in development and will be used for the actual patient data.

## Conclusion

A robotic set-up for the MATRIX-VT study was successfully used to record optical tracked 3D echocardiography phantom data while meeting all requirements for LV motion analysis like frame rate, time synchronization and spatial resolution. Hence, the study is conducted using the same hardware. In addition, a modular motion analysis framework for the evaluation of the MATRIX-VT data was developed. It covers all necessary steps to transfer and evaluate US information in CT coordinates and could thus be utilized as a predictive motion management strategy for STAR therapy. State-of-the-art methods perform well when evaluated independently but the cumulative error exceeds the goal of 5 mm accuracy for extreme probe positions. Several improvement strategies were presented and will be implemented. The next steps include the evaluation of patient data with minor adjustments in calibration and registration methods depending on the actual probe motion during patient examination and the development of an anatomical-based 3D/3D registration method that will allow frame by frame evaluation of the LV contraction.
